# The CRISPR/Cas9 system sheds new lights on the biology of protozoan parasites

**DOI:** 10.1007/s00253-018-8927-3

**Published:** 2018-04-06

**Authors:** Maciej Grzybek, Aleksandra Golonko, Aleksandra Górska, Klaudiusz Szczepaniak, Aneta Strachecka, Anna Lass, Paweł Lisowski

**Affiliations:** 1 Department of Tropical Parasitology, Medical University of Gdansk, Powstania Styczniowego 9b, 81-519 Gdynia, Poland; 2 Department of Biotechnology, Bialystok University of Technology, Wiejska 45E, 15-351, Bialystok, Poland; 3 Faculty of Veterinary Medicine, University of Life Sciences in Lublin, Akademicka 13, 20-950 Lublin, Poland; 4 Faculty of Biology, Animal Sciences and Bioeconomy, University of Life Sciences in Lublin, Akademicka 13, 20-950 Lublin, Poland; 5 Department of Molecular Biology, Institute of Genetics and Animal Breeding PAS, Postepu 36A, 05-552 Jastrzebiec, Poland; 6 iPS Cell-Based Disease Modeling Group, Max-Delbrück-Center for Molecular Medicine (MDC) in the Helmholtz Association, Robert-Rössle-Str. 10, 13092 Berlin, Germany

**Keywords:** CRISPR, Cas9, Genetic modification, Protozoa, Apicomplexa, Genome editing

## Abstract

The CRISPR/Cas9 system, a natural defence system of bacterial organisms, has recently been used to modify genomes of the most important protozoa parasites. Successful genome manipulations with the CRISPR/Cas9 system are changing the present view of genetics in parasitology. The application of this system offers a major chance to overcome the current restriction in culturing, maintaining and analysing protozoan parasites, and allows dynamic analysis of parasite genes functions, leading to a better understanding of pathogenesis. CRISPR/Cas9 system will have a significant influence on the process of developing novel drugs and treatment strategies against protozoa parasites.

## Introduction

### What is CRISPR?

CRISPR (clustered regularly interspaced short palindromic repeats) is a natural part of the genome, found in bacteria and archaea (Barrangou and Marraffini 2014; Westra et al. 2012). It provides adaptive immunity against viruses and plasmids (Jinek et al. 2012) by using CRISPR RNAs (crRNAS) that silence invading nucleic acids (Barrangou et al. 2007; Goldberg and Marraffini 2015; Jiang and Marraffini 2015; Makarova et al. 2011; Mali et al. 2013). It was first observed in the 1980s in *Escherichia coli* (Ishino et al. 1987).

CRISPR means the grouping of short (about 20 bp) palindromic sequences separated from each other on a regular basis that isolates DNA sequences recognised as foreign- so-called “spacers” (Deltcheva et al. 2011). During the adaptation stage, a short fragment from plasmid or viral DNA is incorporated into the CRISPR locus (Datsenko et al. 2012; Yosef et al. 2012). Selection of a sequence that is built into the host genome depends on the presence of the PAM (protospacer adjacent motif) sequence in foreign DNA. PAM is a sequence of several (2–6) nucleotides directly following the DNA sequence that targets the Cas nuclease (CRISPR-associated protein), so the PAM sequence is not incorporated into the genome (it is only a nuclease-targeting element). Expression results in a long, precursor transcript (pre-crRNA) that contains all of the sequences included repeated and separating sequences, which is then matured into the crRNA. CRISPR type II—most common in genetic engineering—is characterised by the presence of trac-crRNA, which is responsible for the maturation of the crRNA (tracRNA hybridises with repeated sequences of pre-crRNA) (Cong et al. 2013). The tracrRNA and pre-crRNA duplex are hydrolysed by endoribonuclease III in the presence of the Cas9 protein. Those products are mature crRNAs containing a spacer and partially repetitive sequence. In the last step (interference), the crRNA directs the Cas9 protein to a complementary, foreign sequence which is then subjected to degradation (Wang et al. 2016a).

**Fig. 1:**
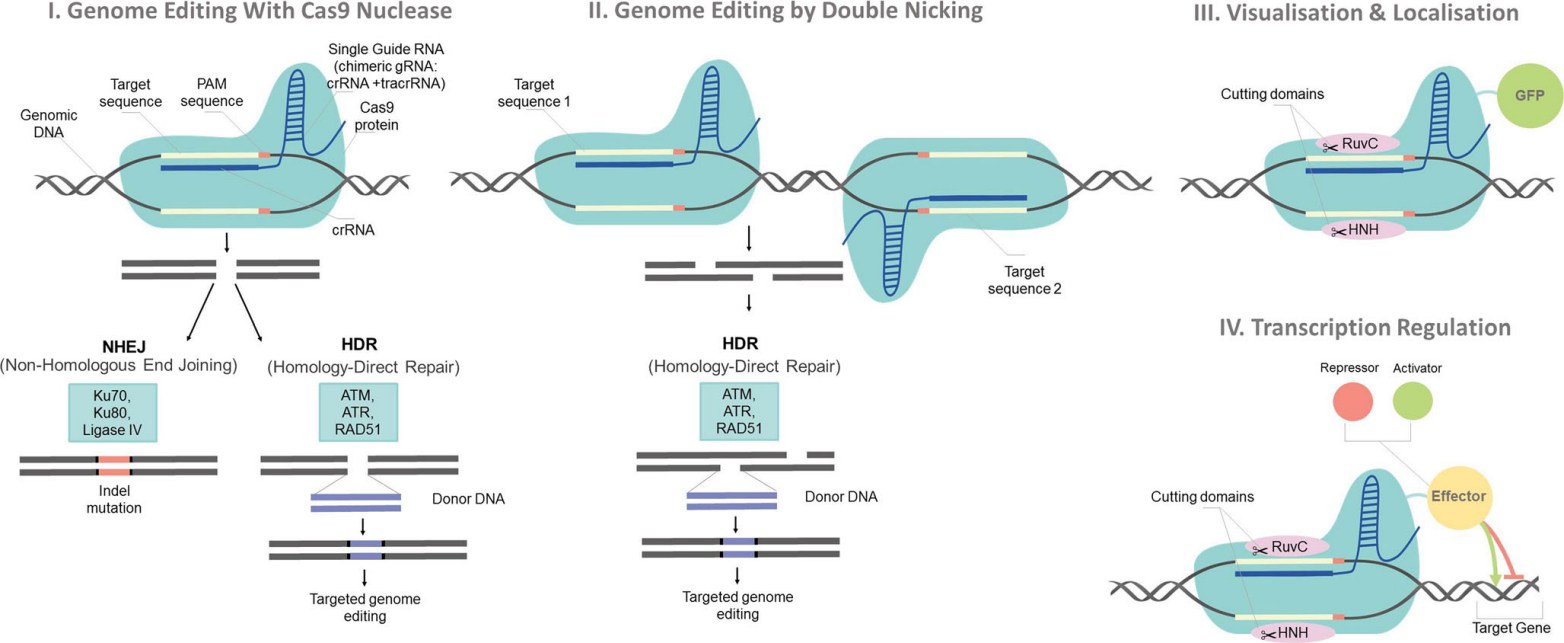
The mechanism of genome editing using CRISPR/Cas9

The activity of the natural CRISPR/Cas9 type II system differs from that used in the genome engineering. To change the genome, it is necessary to provide the Cas9 enzyme and sgRNA (single-guide RNA) scaffold in the form of a plasmid transgene or a complete transcript (Yin et al. 2015). SgRNA is a combination of the crRNA with tracrRNA and is responsible for recognising the target DNA **(**Fig. 1**).** As in the bacterial system, the presence of a PAM directly after the target sequence is required to hydrolyse DNA via cas9. In mammalian cells, the Cas9 nuclease induces the formation of double-strand breaks (DSB) which can be repaired by two primary mechanisms. The first type of repair is a non-homologous end joining (HDR) which occurs more often, but results in the insertion or removal of additional nucleotides, and thus the formation of indel (insertion/deletion) mutations (Lieber 2010). This type of repair depends, among other things, on the activity of DNA ligase IV, heterodimeric Ku protein, DNA-PK_CS_ protein kinase and XRCC4 (Goldberg and Marraffini 2015). The second type of repair—homologous recombination (HDR)—is much less frequent, and in genome engineering is used for precise corrections: insertion (using a DNA template) or deletion of a specific fragment (Zhang et al. 2017). It is also possible to induce genome correction in a particular locus by cutting and inserting the appropriate sequence. To enhance the HDR process and to create precise mutations, the essential proteins of the NHEJ process: Ku70, Ku80 and DNA ligase IV are inhibited by gene silencing or by using inhibitors of these proteins (Chu et al. 2015). The Cas9-sgRNA complex recognises the target sequence of sgRNA-DNA complementarity and by interacting with Cas9 to PAM **(**Fig. 1**)**. Designing and matching a sgRNA sequence of approximately 20 nucleotides to a particular sequence enables the induction of a cut in a strictly defined place in the genome. Also, modifications may involve the structure of the Cas9 nuclease. Cas9 from *Streptococcus pyogenes* is currently widely used in genome engineering and binds to the 5′NGG3 PAM sequence, but this specificity can be modified by orthologous exchange of a PAM-interacting domain in Cas9. For example, orthologous replacement of the Cas9 domain from *Streptococcus thermophilus* with the corresponding domain *Streptococcus pyogenes* exchange the recognised PAM sequence from 5′-NGGNG to 5′-NGG. The Cas9 protein contains two nuclease domains: HNH and RuvC responsible for the cleavage of both strands of DNA (Hsu et al. 2014). Induce of a mutation in one of the nucleic domains allows for the formation of a Cas9 nuclease that cut only one strand, while the mutations in both domains form Cas9 only with sgRNA-guiding activity and binding to the target sequence (Makarova et al. 2011).

CRISPR-Cas9 system is one of the fastest developing areas in bioscience (Doudna and Charpentier 2014). It was successfully applied to edit genomes in a wide range of model organisms including *Drosophila melanogaster* (Gratz et al. 2013), *Saccharomyces cerevisiae* (Dicarlo et al. 2013) and *Caenorhabditis elegans* (Dickinson and Goldstein 2016).

The system has recently been used in a wide variety of biomedical studies including stem cell research (Inak et al. 2017; Yumlu et al. 2017), neurobiology (Grzybek et al. 2017), cancer treatment (Cyranoski 2016), generation of animal models (Mali et al. 2013), somatic genome editing (Schwank et al. 2013), correcting genetics disorders (Long et al. 2016) and the spread and treatment of infectious diseases (Doerflinger et al. 2017; Liao et al. 2015).

The first reports of successful protozoa genome editing using this system were published in 2014 (Ghorbal et al. 2014). However, there is a significant number of studies underline the importance of these tools for genomic engineering (Table 1). These methods became fundamental to the functional assessment of genes (Barrangou and Horvath 2017), and they have significantly advanced our knowledge about parasitosis.

**Table 1:** Applications of CRISPR/Cas9 system in protozoa parasites

Organism	Purpose	Strategy	Repair mechanism	References
*P. falciparum*	Gene knockout	2 vectors	HDR	Ghorbal et al., 2014
*P. falciparum*	Gene knockout	2 vectors	HDR	Wagner et al., 2014
	Gene knockout, C-terminal			
*P. yoelii*	tagging and insertion of point mutations	1 vector	HDR	Zhang et al., 2014
*T. gondi*	Gene knockout and knockin	1 vector	NHEJ, HDR	Shen et al., 2014; Behnke et al., 2015a; Rugarabamu et al., 2015; Wang et al. 2016c
*T. gondi*	Gene knockout, C-terminal tagging and insertion of point mutations	1 vector	NHEJ, HDR	Sidiki et al., 2014
*T. gondi*	Gene tandem replacement	1 vector	HDR	Behnke et al., 2015b
*T. gondi*	Gene knockout	1 vector	NHEJ	Zheng et al., 2015
*C. parvum*	Gene knockout and knockin	1 vector	HDR	Vinayak et al., 2015
*T. cruzi*	Gene knockout	1 vector and 2 vectors	MMEJ, HDR	Lander et al., 2015
*T. cruzi*	Gene disruption (single, multi), exogenous gene swapping	1 vector	MMEJ, HDR	Peng et al., 2014
*T. brucei*	Gene knockin	1 vector	MMEJ	Beneke et al., 2017
*L. major*	Replacement of a gene tandem	2 vectors	HDR	Sollelis et al., 2015, Zhang et al., 2017; Beneke et al., 2017
*L. mexicana*	Gene knockin	1 vector and 2 vectors	MMEJ, HDR	Beneke et al., 2017; Zhang et al., 2017
*L. donovani*	Gene knockout and C-terminal tagging	2 vectors	IHR, MMEJ, HDR	Zhang and Matlashewski (2015); Zhang et al., 2017
*T. vaginalis*	Gene knockout and knockin	1 vector and 2 vectors	NHEJ, HDR	Jannsen et al., 2018
*Giardia intestinalis*				
*Babesia* spp.				
*Eimeria* spp.				
*Isospora* spp.				
*Cystoisospora* spp.				
*Sarcocystis* spp.				
*Naegleria fowleri*			?	
*Entamoeba histolytica*				
*Acanthoamoeba* spp.				
*Pentatrichomonas*				
*hominis*				
*Balantidium coli*				
*Spironucleus* spp.				

In this review, we present findings which demonstrate the recent progress in using CRISPR and CRISPR-associated (Cas) genes. In our opinion, CRISPR/Cas9 system has shed new light on parasitology, especially on protozoa parasites.

## Application of CRISPR/Cas9 system in protozoa parasites

### *Plasmodium falciparum*

*P. falciparum* is the most significant protozoan species causing malaria. The use of genome editing methods, especially CRISPR/Cas9, enables the study of genes involved in the growth of parasites and the invasion process. In the case of *Plasmodium*, it is crucial to know the gene products responsible for the host erythrocyte invasion. A study by Ghorbal et al. (2014) emphasised the significance of the CRISPR/Cas system for *P. falciparum*. Two techniques, namely gene disruption and single-nucleotide gene editing, were used to disrupt two non-essential loci, i.e. the integrated GFP reporter gene and the *kahrp* gene, which encode a protein exported into the host red blood cells. They were acquired by inserting a selectable marker into their encoding sequences. Other complex approaches included the modification of gene sequences *orc1* (origin recognition complex subunit 1), which has a regulatory role in origin activation and *kelch13*—associated with artemisinin resistance*.* Altered parasites were recovered with high efficiency. This was obtained despite the absence of non-mediated range for the change, but only in the case of the cross-transferred plasmids. This might suggest that it is possible to generate neutral or deleterious mutations in the CRISPR system. Similar to previous zinc finger nuclease editing (Straimer et al. 2012), the inclusion of silent mutations in the donor sequences seemingly enriched the desired alterations within the bulk parasite population. As a result, the donor plasmid, as well as the edited genomic locus from cleavage, was protected.

Ghorbal et al. (2014) used a two-plasmid protocol to express both Cas9 nuclease and sgRNA. Considering the need for an appropriate place for sgRNA transcription initiation, the U6 promoter of the spliceosome component of small nuclear RNA (snRNA) is necessary. The U6 transcription of snRNA is driven by RNA polymerase III, which accurately transcribes RNAs that do not encode proteins (non-coding RNAs). U6 product transcription initiates with a G and consists of the motif G-N_19/20_-NGG, with the final three PAM (protospacer adjacent motif) nucleotides essential for cleavage. The products of U6 transcription begin with a guanosine nucleotide (G), and thus the choice of targeting sequences in the genome is limited. The guide sequence (gRNA) pairs with the DNA target, directly upstream of a requisite 5′-NGG adjacent motif. Once the Cas9-gRNA complex binds a putative DNA target, a “seed” sequence at the 3′ end of the gRNA targeting sequence begins to anneal to the target DNA. Cas9 mediates a DSB ~ 3 bp upstream of the PAM.

Ghorbal et al. (2014) reported that the application of the *P. falciparum* U6 snRNA behind the control region made it possible to promote sgRNA expression in the parasite but without the need for the incipient guanosine nucleotide. This is essential as it greatly extends potentially targeted loci in the parasitic genome to random sequences containing the 5′-NGG PAM pattern. It is a particularly beneficial trait of *Plasmodium* spp. with a mean GC content of just 19.4%.

*Plasmodium* spp. lacks the mechanisms necessary for non-homologous end joining (NHEJ) (Straimer et al. 2012). Therefore, the use of the CRISPR/Cas compound, i.e. rift followed by error-susceptible fixing to produce frame displacement mutations, is not an option to malaria investigators. Nonetheless, there are numerous possibilities, such as the ability to express two or more gRNAs within the same cell. This might permit multiple cleavage instances that can produce substantial deletions. These can be used to analyse genome duplications that might be connected resistance to drugs. Work by Gilbert et al. showed that a version of Cas9 without endonuclease activity (catalytically dead Cas9; dCas9), delivered to a target gene, acts as a blocker to transcription. On the other hand, gene expression can be increased by the merging of this non-active Cas9 with a transcription-triggering domain (Gilbert et al. 2013). Mutations in pfmdr1 gene are associated with *P. falciparum* resistance to drugs, e.g. the point mutation in codon 86 pfmdr1 is associated with decreased sensitivity to chloroquine, while amplification of this gene is associated with resistance to antimalarials (i.e. mefloquine and halofantrine) (Antony et al. 2016).

Another study applied CRISPR/Cas9 to edit *P. falciparum* genome (Wagner et al. 2014). They created a two-vector strategy to apply T7 promoter-driven system. The team used *pT7 RNAP-HR* plasmid to express *T7 RNA polymerase* and to deliver DNA donor sequence for DSB repair. The second *pCas9-sgRNA-T* plasmid was created to express Cas9 and sgRNA to target locus of interest. With the application of the system, authors reported *kharp* gene deletion 33 days post-transfection. What is more, the team managed to delete *eba-175* gene, which is responsible for encoding parasite ligand. The editing efficiency between 50 and 100% was reported. This confirms that CRISPR/Cas9 system may be applied to edit *Plasmodium* spp. genome successfully.

Recent reports have underlined the reality of significant resistance to first-line antimalarial combination therapies. The main aim of the pharmacologists and parasitologists working on malaria is to increase treatment efficacy (WHO 2015). Increasing resistance to artemisinin and piperaquine has been reported recently (Amaratunga et al. 2016; Isozumi et al. 2015), and this underlines the gravity of the situation. Ng and colleagues applied a similar strategy to Wagner et al. (2014). They used whole-genome sequencing and the CRISPR/Cas9 system to ratify the role of PfMDR1 (*Plasmodium falciparum* multidrug drug resistance gene 1) mutations in resistance to ACT-451840, a compound that contains piperazine and can affect *Plasmodium* asexual development occurring in the blood (Ng et al. 2016). The piperazine-rich compound ACT-451840 was found by the authors to promote inhibition of asexual stages and transferrable gametocyte forms of *P. falciparum*. Genomic sequencing of in vitro-derived ACT-451840-resistant parasites found SNPs (single nucleotide polymorphism) in *pfmdr1*. It encodes a digestive vacuole membrane-bound ATP-binding cassette transporter responsible for altering *P. falciparum* receptiveness to various first-line pharmaceuticals used against malaria. The CRISPR/Cas9-associated editing of genes enhances the promotion of ACT-451840 resistance by PfMDR1 point mutations. Resistant parasites showed an improved response to clinically applied anti-malaria drugs. *pfmdr1* mutation obtained via Cas9 caused resistance to ACT-451840 in fifth stage gametocytes. This proves that PfMDR1 mutations can lead the resistance to asexual sanguineous stages and mature gametocytes by developing a response to active compounds. The investigation of PfMDR1 resistance mechanisms opens up new vistas in the development of new antimalarial pharmaceutic agents (Ng et al. 2016).

**Fig. 2:**
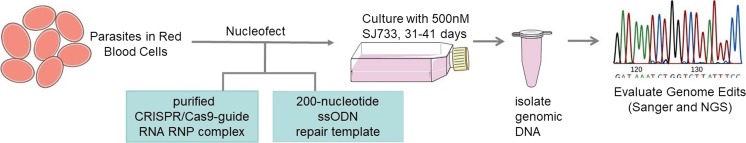
Strategy for introducing plasmid-free CRISPR/Cas9 edits to the *P. falciparum* gene pfatp4 without the use of plasmids. Adopted from Crawford et al. 2017

A most recent study by Crawford et al. (2017) used CRISPR/Cas-9 to introduce a drug resistance mutation in *Plasmodium*. This novel attempt does not require the use of plasmids or cloned homologous recombination templates. The team edited PfATP4, a sodium efflux channel by CRISPR modified RNA-protein complex and single-stranded oligodeoxynucleotide repair mechanism. Whole-genome sequencing (WGS) and inhibition assay methods confirmed that obtained mutation caused the development of resistance to an SJ733 compound (Fig. 2) (Jimenez-Diaz et al. 2014).

What is more, authors showed that single-stranded oligodeoxynucleotide might play a role of repair template in *P. falciparum*. Obtained results demonstrate that CRISPR/Cas-9 system can be successfully applied to edit *Plasmodium* genes to study malaria drug resistance and therefore search for new antimalaria treatment in the future.

### *Toxoplasma gondii*

*T. gondii* is an obligate, intracellular parasite in humans and animals (Tenter et al. 2000). This species is also used as a model for other apicomplexan parasites including *Plasmodium* spp. (Wang et al. 2016b). Despite the recent favourable developments in molecular techniques, manipulating the *T. gondii* genome is still not efficient (Szabo and Finney 2017). Moreover, manipulations are restricted to strains or lines carrying mutations that allow selection. What is more, one of the *T. gondii* features is a highly clonal population structure, and these populations differ in acute virulence in laboratory mice. Establishing a link between the extent of virulence factors and pathogenesis is a serious issue.

In their study, Shen et al. (2014) used the CRISPR/Cas9 system to disrupt *T. gondii* genes efficiently. With a combination of site-specific sgRNAs and Cas9, they disrupted the uracil phosphoribosyltransferase (*UPRT*) gene which builds resistance to fluoro deoxyribose (FUDR). This permitted straightforward efficiency quantification and provided a facilitative site for analyses of gene disruption frequencies. Moreover, Shen et al. (2014) applied the CRISPR/Cas9 system to edit the ROP18 (gondii rhoptry protein) locus from the type 1 strain GT1 to complement this loss at the UPRT locus. This revealed that ROP18 is a significant factor that influences the acute virulence and confirmed previous hypotheses (Fentress et al. 2010).

**Fig. 3:**
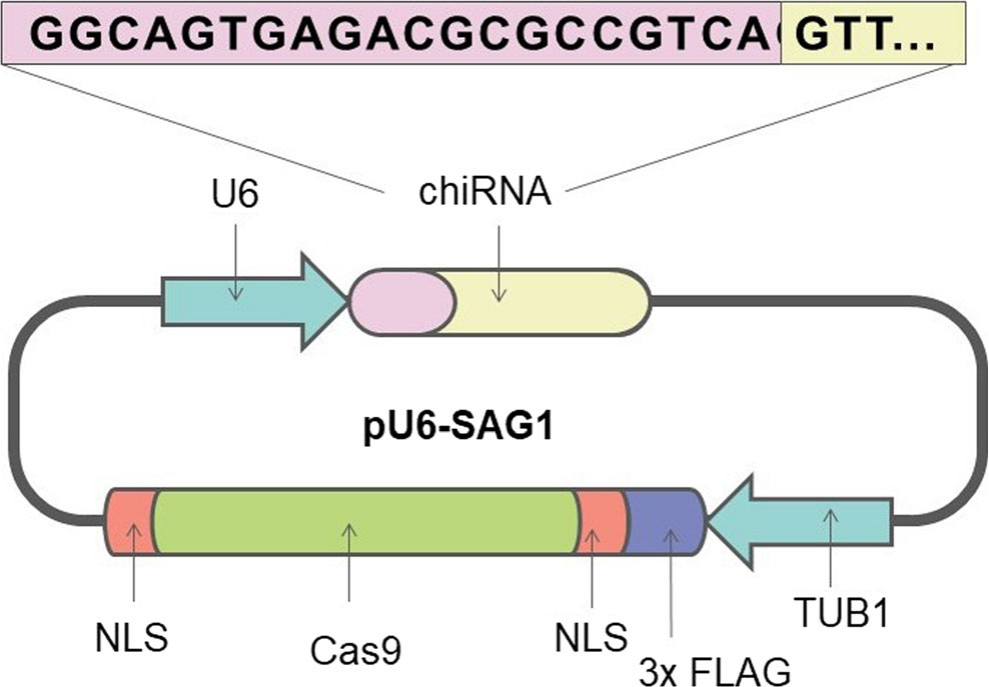
Targeted disruption of the SAG1 locus using CRISPR/Cas9. Adopted from Sidik et al. 2014

Ku80 and Ligase IV DNA are key molecules in the NHEJ repair pathway, so their inhibition improves the efficiency of the competitive HDR pathway. The recent generation of NHEJ deficient strains via the elimination of KU80 has made genome editing available. This is possible due to the suppression of the high incidence of accidental integration, naturally occurring in *T. gondii.* However, this method makes use of complex DNA constructs for homologous recombination. This gives rise to many problems such as the necessity of screening many clones for correct rearrangements. Moreover, genomic engineering predominantly concerns chosen, laboratory-adapted strains of the *ΔKU80 T. gondii* lines mentioned previously. A recent study by Sidik et al. (2014) reported an efficiently edited *T. gondii* genome via the CRISPR/Cas9 system and solved the above issues. The team used the RNA-guided Cas9 nuclease for the generation of non-selection knockouts. The author introduced point mutations and epitope tags into the parasite genome. Sidik and colleagues confirmed that Δ*KU80 T. gondii* is more susceptible to genetic edition than wild-type. This is caused by a lack of NHEJ machinery for double-strand breaks repair. The authors have previously reported highly efficient genome editing allowing the introduction of point mutations and epitope tags into the *T. gondii* genome (Fig. 3). Another study by Sidik et al. (2016) applied the CRISPR/Cas9 system to conduct the first genome-wide genetic screening to analyse the contribution of every single gene of *T. gondii* during infection of human fibroblasts. The study identified significant numbers of novel genes, responsible for infection process, unique to the phylum. Secondary screening identified the claudin-like apicomplexan microneme protein (CLAMP) as an invasion factor. This will help to understand parasite metabolic needs, host specificity or resistance to drugs. Moreover, proposed protocols enable to perform the screening within a short (around 3 weeks) period of time (Sidik et al. 2018).

Sequenced apicomplexan genomes contain CLAMP throughout the genome. It performs a crucial role in asexual *P. falciparum* stages. These findings offer comprehensive functional data on *T. gondii* genes which will help to promote further attempts at broadening the general perspective on interventions aimed at parasite containment.

Post-transcriptional control of gene expression in protozoa parasites is still not fully understood. Nucleo-cytoplasmic RNA export is a crucial step in gene expression control. UAP56 protein, the most studied splicing factor, has various functions in the pre-spliceosome assembly and mRNA nuclear export. Serpeloni et al. (2016) studied TgUAP56, a component of mRNA export machinery using *T. gondii* as a model organism and CRISPR/Cas9 as an editing tool. The CRISPR/Cas9 system may be applied to monitor mRNA export factors according to the functional genomic approach by disrupting expression of selected genes by ddCas9 (fusion of ddFKBP with Cas9). Researchers applied the qualified Cas9/CRISPR system to perform a genetic screening to check whether a given parameter participated in mRNA exportation in *T. gondii*. The results showed the TgRRM_1330 protein led to the aggregation of mRNA in the nucleus as observed in the case of TgUAP56. This study is a perfect example of CRISPR/Cas9 system application for studying molecular processes in parasitic protozoa. The obtained knowledge lets us explore another mRNA export path in apicomplexans.

### *Cryptosporidium parvum*

Recent reports have underlined the role of *Cryptosporidium* spp. as a significant diarrhoeal pathogen in children (Checkley et al. 2015; Kotloff et al. 2013; Mondal et al. 2012; Striepen 2013) As an apicomplexan, this species has a deficient number of genes (~ 3950 genes) in its genome. A multiple intestinal life cycle stage, weak tractability of the parasite because of inefficient culture methods and lack of animal models that insufficiently imitate the development cycle have all contributed to the fact that no vaccine against cryptosporidiosis is currently available. Furthermore, there is only one approved drug available—nitazoxanide—but it is not a cure for all of the adverse effects of this pathogen.

This situation may change significantly due to a recent achievement published by Vinayak et al. (Vinayak et al. 2015). An optimisation of known genetic modification methods led to successful transient transfection of *Cryptosporidium* sporozoites. The authors have established and optimised the transfection of *C. parvum* sporozoites in tissue culture in HCT-8 cells. Transfection was done by excystation of sporozoites from oocysts obtained from experimentally infected cows. Applied protocol allowed to mimic the parasite intestinal phase (Gut and Nelson 1999). The use of CRISPR/Cas9 technology has allowed detection of genes suspected of responding to neomycin resistance. To build a *C. parvum* CRISPR/Cas9 system, they constructed a plasmid where Cas9 gene was flanked by parasite regulatory sequences, and the luciferase gene was deactivated. Luciferase activity was restored when *C. parvum* sporozoites were cotransfected with a specific guide. Also, translational fusions between the Nluc reporter and the neomycin resistance marker (Neo) were constructed to focus observation on the small subset of transfected parasites.

The authors introduced a plasmid-encoding target gene whose short expression occurs in the cell. The verification of successful transfection was performed using a gene that encodes luciferase. It lights up when the appropriate substrate is present. This marker is linked to a gene which confers resistance to neomycin-class antibiotics, this, in turn, provides a means of selecting transferred cells.

**Fig. 4:**
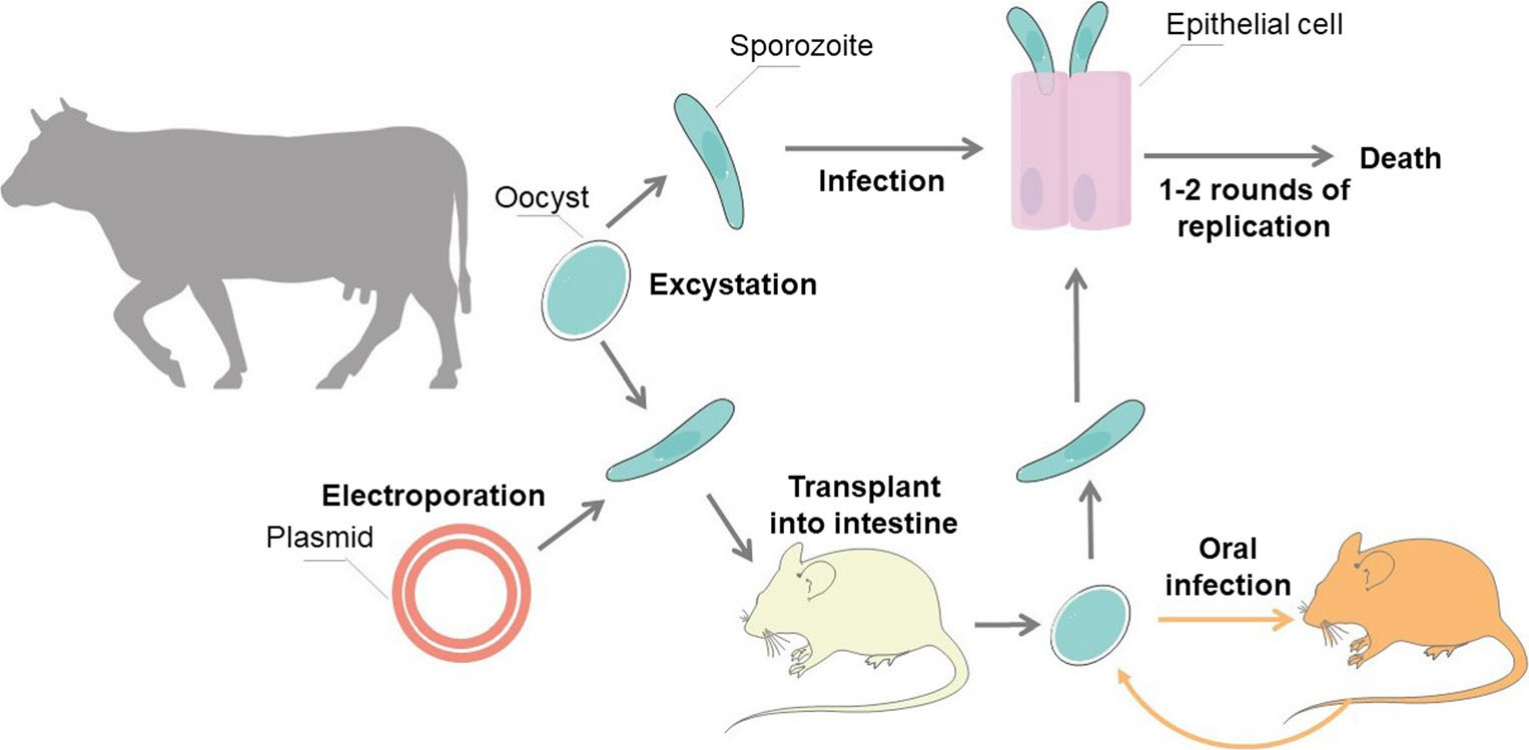
Modification and culture of *Cryptosporidium.* Adopted and reproduced from Vinayak et al., 2015

*Cryptosporidium* does not produce oocysts when cultured in vitro. However, this issue was solved by placing engineered sporozoites back into intestines of immunodeficient mice, from which oocysts were collected (Fig. 4).

A significant problem with *Cryptosporidium* culturing in vitro was overcome using the CRISPR/Cas9 system*.* The cross-transfer of sporozoites with both the luciferase-neomycin resistance fusional gene and DNA-coding CRISPR/Cas9 mechanism, preceding the sporozoite infections and paromomycin treatment of the mice, allows for the retrieval of antibiotic-resistant parasites from mouse faeces. The parasites obtained reliably expressed an integrated luciferase gene. The application of CRISPR/Cas9 system permit genetic modification and culturing (Pawlowic et al. 2017) of this significant parasite and will hopefully bring rapid advances in overcoming cryptosporidiosis.

### *Trypanosoma* spp.

*Trypanosoma cruzi* is a protozoan parasite that infects both humans and animals. It affects more than 10 million people (Jones et al. 2008). It is the causative agent of Chagas’ disease. *T. cruzi* is the least well-understood protozoan in a group of neglected tropical diseases, being responsible for infection-induced heart disease. The complexity of *T. cruzi* genetics and the lack of efficient biomolecular tools for genome editing have created a significant barrier to the investigation of this organism.

However, this situation might be changing given the recent findings of Peng et al. (2014), who adapted the CRISPR-Cas9 system for quick and efficacious suppression of numerous endogenous genes, including crucial *T. cruzi* genes. Researchers have designed sgRNAs targeting the α-tubulin and histidine ammonia lyase (HAL) genes, respectively, while the ability of sgRNA to effectively disruption of gene expression was determined by the loss of GFP expression in the parasites studied. The disappearance of a template effectuating the fixing of the Cas9-incited DSBs in *T. cruzi* is brought about only through microhomology-mediated end joining (MMEJ) (Glover et al. 2008, 2011) with deletions of different sizes. This DNA repair template provided homologous recombination which led to DSB repair with efficiency several times higher than it is without CRISPR-Cas9-induced DSBs. What is more, Peng et al. demonstrated the significant multiplexing capability of CRISPR-Cas9 in *T. cruzi* by suppressing the expression of a sizeable family of enzyme genes (β-galactofuranosyl glycosyltransferase family). This led to a substantial diminishment of the enzymatic product with no visible mutations off the target.

**Fig. 5:**
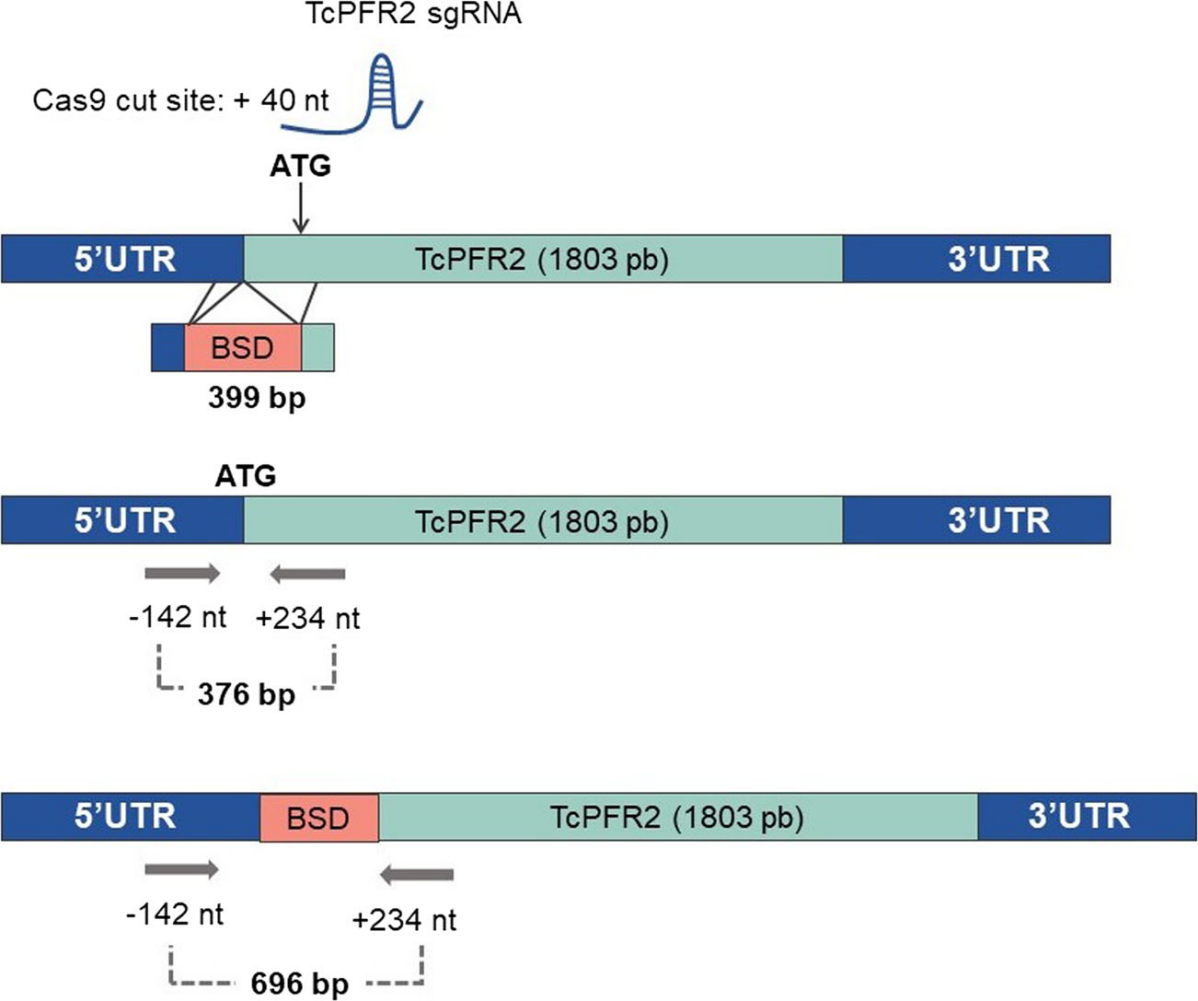
Generation of a TcPFR2-KO mutant by CRISPR/Cas9-induced homologous recombination. A DS gDNA break was obtained by Cas9 at nt +40 of the TcPFR2 open reading frame. DNA was repaired with a linear blasticidin S deaminase cassette containing 100-bp homologous regions from the TcPFR2 locus. Adopted from Lander et al. 2015

Lander et al. (2015) performed three different types of *T. cruzi* gene disruption using the CRISPR/Cas9 system. The team silenced GP72 protein genes—connected with infectivity—which is necessary for correct flagellar attachment by using vectors containing sgRNA and Cas9. Moreover, significant components of the parasite flagellum such as PFR1, PFR2 were silence as well. Lander et al. used a vector that contained sgRNA and Cas9 as well as donor DNA for homologous recombination to create cell lines with the following modified genes: *PFR1*, *PFR2* and *GP72* (Lander et al. 2015) (Fig. 5). This confirmed that the CRISPR/Cas9 system might be applied to modify the *T. cruzi* genome without the adverse effects of Cas9 toxicity. What is more, the PFR1, PFR2 and GP72 genes are responsible for the motility of the parasite.

Another study, by the same group, reports application of CRISPR/Cas9 system in tagging genes of *T. cruzi*. Results confirm that genes of known (flagellar calcium bining (TcFCaB), vacuolar proton pyrophosphatase (TcVP1) and unknown locations (mitochondrial calcium uniporter (TcMCU) and inositol 1,4,5-trisphosphate receptor (TcIP_3_R)) may be tagged using CRISPR/Cas system. This may be applied to characterise locations and functions of *T. cruzi* proteins (Lander et al. 2016b). Moreover, formation of a protocol to generate CRISPR/Cas9-mediated endogenous gene tagging will enable analysis of protein–protein interactions in *Trypanosoma* spp. (Lander et al. 2017). This novel approach may be applied in both *T. cruzi* and *T. brucei* to study targets for drugs or vaccines against kinetoplastid parasites (Chiurillo et al. 2017). CRISPR application caused an improvement in these parasite genome functional analyses (Lander et al. 2016a). These results have established a robust novel tool for the analysis of the gene functions of *Trypnosoma* spp., a barely genetically tractable parasite.

### *Leishmania* spp.

*Leishmania* spp., a protozoan parasite, causes fatal human visceral (VL), cutaneous (CL) and mucocutaneous (MCS) leishmaniasis. These obligate parasites of host macrophages are transferred by over 30 various species of phlebotomine sandflies (Pavli and Maltezou 2010). Leishmaniasis constitutes the second most problematic infectious disease the world over, present in more than 98 countries, threatening over 350 million people (Ejov and Dagne 2014).

Genetic manipulations are problematic in leishmaniasis species since multiple cloning and transfection steps are necessary to generate null mutants for targeted genes. However, a recent application of the CRISPR/Cas9 system enables highly efficient *Leishmania* genome editing. Sollelis et al. (2015) expressed Cas9 endonuclease controlled with the following promoter: dihydrofolate reductase–thymidylate synthase (DHFR-TS). Subsequently, the single-guide RNA was generated under the control of the promoter and terminator U6snRNA. The team targeted the paraflagellar rod-2 locus, a tandemly repeated gene family. What is more, the authors obtained null mutants in a single round of transfection. Entire genome sequencing of two independent clones confirmed the absence of off-target editions.

Another study applied CRISPR/Cas9 system into *L. donovani* and successfully produced guide RNA expression vectors through the *L. donovani* rRNA promotor and ribozyme of the hepatitis delta virus. Zhang and Matlashewski (2015) were able to produce knockout with no selection through the insertion of an oligonucleotide donor containing stop codons as well as 25-nucleotide homology arms in the Cas9 cleavage site. They disjointed and accurately marked endogenous genes through the insertion of a bleomycin drug choice marker and reporter gene (GFP) in the Cas9 cleavage site. Hammerhead and HDV ribozymes are self-processed from the gRNA they were employed to create a double-gRNA expression vector to express two gRNAs at the same time. This application caused the deletion of the 3-kb miltefosine transporter gene (LdMT) what cause improvement in gene inactivation efficiency. The authors report a novel single point mutation in LdMT (M381T) that was linked to miltefosine resistance. This was a concern for the only available oral treatment against leishmaniasis. Most importantly, the authors report that *L. donovani* chiefly used HDR and MMEJ to fix the Cas9 nuclease-generated DSB. The NHEJ path seems to be absent in *L. donovani*. These observations corroborate the view that the CRISPR-Cas9 system constitutes an efficient genome engineering instrument for *L. donovani.* The successful application of the CRISPR/Cas9 system (i.e. production of gene knockouts) confirms that it is a useful and multifunction tool for leishmaniasis research.

**Fig. 6:**
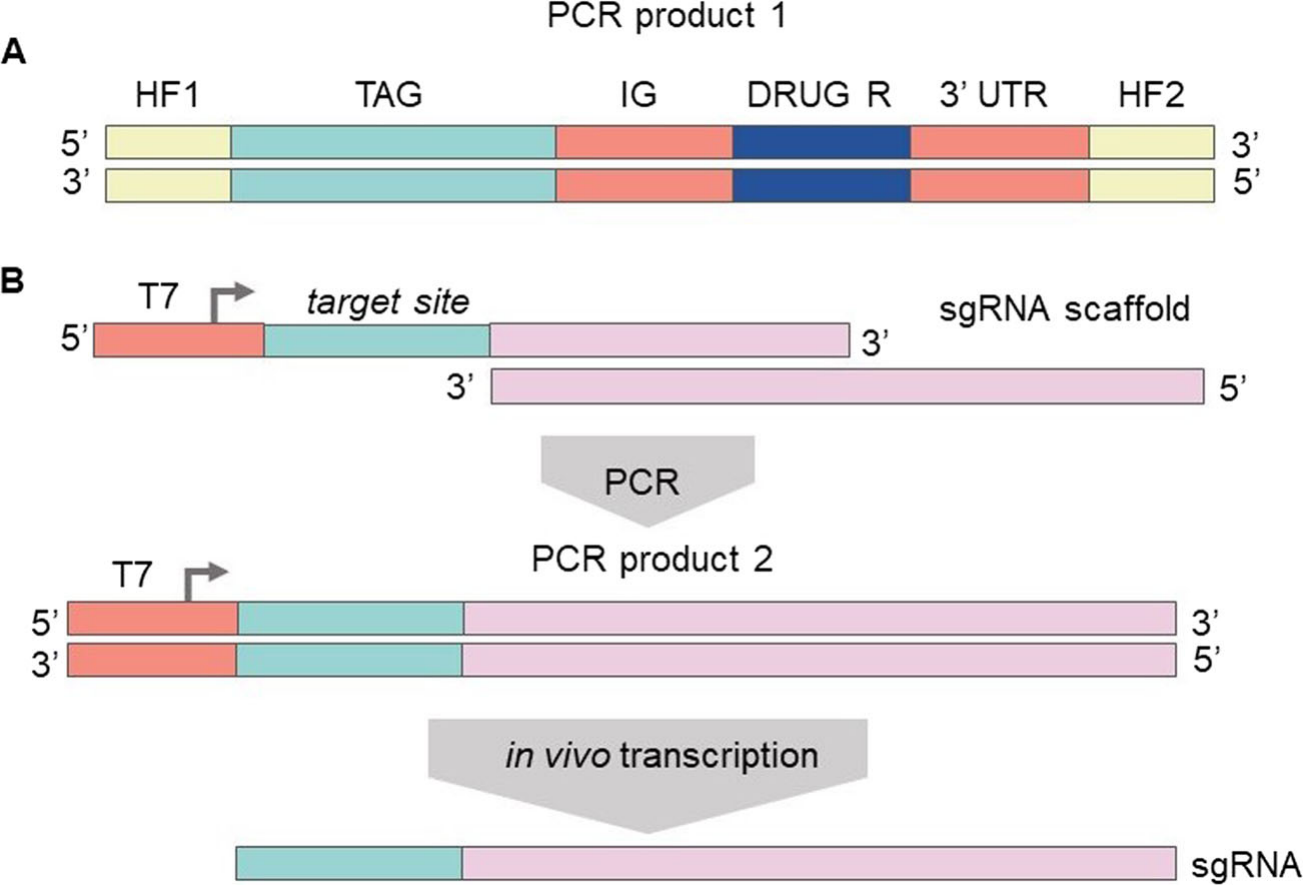
Co-transfection of two PCR amplicons allowed precise insertion of marker genes. PCR-amplified donor DNA containing 30 nt HF specific to the target locus, a fluorescent protein tag and a drug-selectable marker gene. Adapted from Beneke et al. 2017

A most recent study by Beneke et al. applied CRISPR/Cas9 system technology in Leishmania and presented a toolkit that allows the tagging and disruption of parasite genes. The authors bypassed cloning step in bacteria by generating sgRNA and repair DNA by PCR (Fig. 6).

Presented protocols are a significant step in editing kinetoplastids genomes and provide excellent tools to perform large-scale knockout screens (Beneke et al. 2017). Moreover, development of easy to perform and rapid methods for assessment of gene functions (Soares Medeiros et al. 2017) will contribute to decrease of costs and time in developing new successful therapies against kinetoplastid parasites.

### *Trichomonas vaginalis*

*Trichomonas vaginalis* is a pear-shaped, motile parasitic protozoan (Johnston and Mabey 2008). Trichomoniasis is one of the most prevalent sexually transmitted disease (Poole and McClelland 2013), with more than 174 million new cases worldwide (Huppert 2009). The parasite causes the vaginal pH increase and epithelial breaks and inflammation via toxication (Petrin et al. 1998).

A complex application of CRISPR/Cas9 system in *T. vaginalis* was reported recently by Janssen and colleagues (2018). In vivo expression of Cas9 is known to cause problems related to its toxicity (Peng et al. 2014). However, experiments with *T. vaginalis* shown that Cas9 toxicity may be decreased by regulation of FKBP-DD and Shield-1 expression. Low concentration of Shield-1 caused a significant increase in stabilisation of FKBP-Cas9 protein. A significant issue in homologous gene replacement in *T. vaginalis* is caused by low transfection efficiency. The team used nucleofection (Burkard et al. 2007) to increase transfection efficiency. The application of nucleofection causes a 20-fold enhancement of transfection efficiency. It makes able to introduce multiple DNA elements into a single cell of the parasite. Authors proposed a protocol for CRISPR/Cas9-directed homologous recombination that enables to knockout genes, what is a *novum* in *T. vaginalis* genome editing (Brás et al. 2013; Land et al. 2004). Using CRISPR-Cas9-mediated homology repair, they knockout *ferredoxin-1* and *mif* genes. Presented molecular methods for modification of *T. vaginalis* genome allow to study gene function and therefore pathogenesis of this parasite.

### What is next?

CRISPR-Cas systems are *tour de force* achievement in molecular parasitology. CRISPR-Cas system genome manipulations are efficient and should contribute to the discovery of new treatment possibilities. CRISPR-Cas system may be applied to generate immunogenic and non-pathogenic parasites that in the future can serve as immunisation or vaccination (Hollingdale and Sedegah 2017). What is more, validation of clinical and population studies obtained by genome-wide association studies will help with the development of new antiparasitic drug targets (Visscher et al. 2017). CRISPR-Cas system may be used to create libraries applied in drug mechanism research. The CRISP/Cas system might be used for the investigation of genes participating in infection processes. These methods will ameliorate the functional analysis of parasite genes which enables the effective engineering of their genomes. What is more, the development of an inducible system allowing the targeting of essential genes is highly significant and urgent (Kostro et al. 2015). It is now entirely credible to assert that protozoa parasites have joined the CRISPR/Cas9-based technologies and there is hope that this system will be applied in other parasitic species (Table 1), such as *Giardia intestinalis*, *Eimeria* spp. or *Babesia* spp. in the nearest future.
